# Right ventricular function in dilated cardiomyopathy: relation to location of focal myocardial fibrosis

**DOI:** 10.1186/1532-429X-15-S1-E120

**Published:** 2013-01-30

**Authors:** Mohamed A Abdelrazek, Florian Andre, Fabian aus dem Siepen, Maria Fernanda Braggion Santos, Evangelos Giannitsis, Hugo A Katus, Hassan Abdel-Aty

**Affiliations:** 1 Universitatsklinikum Heidelberg, Heidelberg, Germany; 2 Radiology, Cairo university, Faculty of Medicine, Cairo, Egypt; 3 School of Medicine of Ribeirao Preto University of Sao Paulo, Sao Paulo, Brazil

## Background

Severe right ventricular dysfunction (RVD) is an important prognostic parameter in dilated cardiomyopathy (DCM). The relationship between myocardial fibrosis of the left ventricle (LV) and particularly the septum and right ventricular (RV) function remains poorly understood. We sought to investigate this relationship in a large cohort of DCM patients using cardiovascular magnetic resonance (CMR).

## Methods

We studied 257 patients (52±15ys,193men) with known DCM using a clinical 1.5 T CMR scanner (Philips Achieva). Short axis slices covering entirely both ventricles were acquired using standard SSFP-sequences for measurement of LV and RV volumes and ejection fraction (EF). Focal myocardial fibrosis was assessed in late gadolinium enhancement (LGE) CMR images acquired 10 minutes after i.v. injection of 0.2mmol/kg Gd-DTPA (Magnevist). SSFP and LGE CMR images were assessed by different observers who were blinded to measurement results and clinical data. Severe RVD was defined as RVEF<35%. In a subgroup of patients (n=96) invasive measurement of pulmonary artery pressure was performed.

## Results

Mean LVEF was 35±14% while mean RVEF was 42±13%. Focal myocardial fibrosis was present in 92 (36%) patients and was located a) intra-murally within the inter-ventricular septum in 29 patients (LGE -septum) or b) sub-epicardially/diffuse in 63 patients (any-LGE). None of the patients had sub-endocardial LGE. LGE-septum patients exhibited a significantly higher RVEF (49±12%; vs. 40±14%;p=0.006), a higher RV EDV index (115±43 ml/m2; vs. 99±23 ml/m2 ;p=0.05) and a higher RV ESV index (73±45 ml/m2; vs. 52±22 ml/m2 ;p=0.01) compared to the patient group with `any-LGE`. LVEF was not significantly different (30±13%; vs. 40±14% p=0.006) between the two LGE-groups. Fifty-five (21%) patients had severe RVD (<35%RVEF) with a mean RVEF of 24±8%. The presence of myocardial fibrosis (septal or non-septal) was not significantly associated with severe RVD (p= 0.23). Also, there was no age and gender dependency in RVD. Invasively, RVD patients had significantly higher pulmonary artery pressure (35±12 mmHg; vs. 29±9 mmHg p=0.03). RV EDV index correlated significantly with pulmonary artery pressure (r= 0.43, p<0.0001).

## Conclusions

The presence of LGE indicating focal fibrosis is not related to severe RV dysfunction in DCM. Interestingly, patients with ventricular septal fibrosis, i.e. mid wall sign, exhibited superior RV function. This may be explained by the pronounced mechanical shear stress exerted by the well-functioning RV on the inter-ventricular septum, potentially inducing focal myocardial fibrosis in DCM patients.

## Funding

none

